# The value of Phosphohistone H3 as a cell proliferation marker in oral squamous cell carcinoma. A comparative study with Ki-67 and the mitotic activity index

**DOI:** 10.4317/medoral.25420

**Published:** 2022-08-17

**Authors:** Natalia Tancredi-Cueto, Gabriela Vigil-Bastitta, Ronell Bologna-Molina, Verónica Beovide‑Cortegoso

**Affiliations:** 1 DMD. Master Student (Dentistry). Oral Histopathology Laboratory, Faculty of Dentistry, University of the Republic, Uruguay; 2 DMD. Master Student (Dentistry). Molecular Pathology Area, Faculty of Dentistry, University of the Republic, Uruguay; 3 PhD. Molecular Pathology Area, Faculty of Dentistry, University of the Republic, Uruguay; 4 PhD. Oral Histopathology Laboratory, Faculty of Dentistry, University of the Republic, Uruguay

## Abstract

**Background:**

The Phosphohistone H3 (PHH3) antibody is recognized as a biomarker of cell proliferation, specific for cells in mitosis, of prognostic value in different malignant neoplasms, however it has been poorly studied in oral squamous cell carcinoma (OSCC). The main objective of this study was to evaluate the immunoexpression of the PHH3 in the OSCC, through the correlation with the immunoexpression of Ki-67, the mitotic activity index (MAI), histological grading, clinical-morphological parameters and the rate of survival.

**Material and Methods:**

The study sample consisted of 62 cases of OSCC diagnosed in the Pathological Anatomy Laboratory of the Faculty of Dentistry, University of the Republic (Uruguay). In each of them, an immunohistochemical technique was performed for Ki-67 and PHH3 (serine 10) antibodies. Image J software was used for the MAI and biomarker quantification, defining the percentage of positivity and mitotic Figures per 1000 tumor cells.

**Results:**

a significant association was obtained between the expression of PHH3 (*p* 0.016) and MAI (*p* 0.031) with survival time. However, no similar relationship was found with Ki-67 (*p* 0.295). Although it was confirmed a statistical association between histological grade and Ki-67 immunoexpression (*p* 0.004), PHH3 did not show a similar relationship (*p* 0.564).

**Conclusions:**

It was confirmed the role of the PHH3 antibody as a biomarker of mitotic Figures in OSCC and as a potential marker of cell proliferation. It is noteworthy that this is one of the first works that evaluates a possible relationship between the expression of this antibody and survival in OSCC.

** Key words:**Oral squamous cell carcinoma, phosphohistone H3, Ki-67, cell proliferation.

## Introduction

Oral squamous cell carcinoma (OSCC) represents the most common cancer of head and neck region (1,2). It is characterized by aggressive biological behavior and an unfavorable prognosis (3,4). In order to improve the low survival rate of patients with OSCC, an important line of research is focused on the identification of molecular biomarkers of prognostic value that contribute to the selection of the most appropriate therapeutic plan (3,5,6).

Cell proliferation is an essential biological process, key in the growth and maintenance of tissue homeostasis, whose loss of control plays a fundamental role in the development of malignant neoplasms (4,6). In fact, in several types of human cancer, the evaluation of cell proliferation is considered an important histological parameter (7). For its assessment, different molecular antigens have been identified, which, when expressed by cells in active proliferation, can be used as predictive biomarkers of sustained proliferation (8). MAI and Ki-67 immunoexpression are the most widely used methods to determine tumor proliferative capacity, however, both methods are objected to present significant intra and interobserver variability (9).

MAI represents the oldest method for determining the proliferative capacity of malignant neoplasms (10,11). It is obtained by counting normal and atypical mitotic Figures (MF) in a cell population of known number (10). The Ki-67 antigen is a non-histone nuclear protein present in all active phases of the cell cycle (G1, S, G2 and M), being absent only in resting cells (G0) (4,9,12). The Ki-67 antibody by immunohistochemical techniques (IHC) allows the identification of the Ki-67 protein in tissue samples (4,12,13). Actively proliferating cells immunoexpress this antibody, varying in intensity and location, depending on the phase of the cell cycle and the history of each individual cell (14,15). The quantification of Ki-67 immunoexpression is performed through the labeling index, defined as the percentage of positive cells in a cell population of known number (3,4,12).

The PHH3 antibody is recognized as a biomarker of cell proliferation, specific for cells in mitosis, by identifying phosphorylated histone H3 by IHC (9,11,16). Histone H3 is one of the five different types of histone proteins that are part of nucleosomes, whose phosphorylation at the serine 10 and 28 level, determines the compaction of chromatin during cell division and from this event the cell is enabled to enter the M phase of the cell cycle (11,16,17). Histone H3 phosphorylation is initiated non-randomly in pericentromic heterochromatin in late G2 phase and as mitosis progresses, it spreads throughout the chromosome, completing in late prophase and continuing through metaphase (11,16). In anaphase, the dephosphorylation process of histone H3 begins, which ends in early telophase (9,11,16). The PHH3 antibody is characterized by presenting a clear and well contrasted immunostaining, limited to cells that are in the M phase of the cell cycle, while interphase cells do not express it or do so minimally (9,11,18). Although it has been recognized as a prognostic marker in multiple types of cancer, it has been scarcely analyzed in OSCC and, in particular, its relationship with survival rate has not been the subject of research until now (16,19).

The main objective of this study was to evaluate the immunoexpression of PHH3 in OSCC, through the correlation with the immunoexpression of Ki-67, MAI, histological grading, clinical-morphological parameters and the rate of survival.

## Material and Methods

The study sample consisted of 62 cases of OSCC, corresponding mostly to incisional biopsies diagnosed in the Pathological Anatomy Laboratory of the University of the Republic (UdelaR) School of Dentistry, in the period 2007-2015. The clinical‑pathological records of each of the cases were reviewed, recording the variables corresponding to gender, age, topography, date of pathological diagnosis and histopathological diagnosis. Survival was defined as the time elapsed between the initial histopathological diagnosis and death from cancer. The histopathological diagnosis of each of the cases was made according to the WHO classification (2017) for OSCC, in well, moderately and poorly differentiated (1,2).

Hematoxylin-eosin (H-E) and IHC histological slides were digitized with the Motic VM 3.0 Digital Slide Scanning System for image acquisition and processing. Motic VM 3.0 Motic Digital Slide Assistant software (version 1.0.7.46, Copyright Motic China Group Co., Ltd.2017) was used for its analysis. Cell quantification was performed with the Image J software manual counting tool (1.52v, Wayne Rasband, National Institutes of Health, USA). For the calculation of the MAI, in the H-E slides, normal and atypical MF present in 1000 tumor cells were counted. The identification of the MF was carried out according to the morphological characteristics described by Van Diest *et al*., recognizing as atypical mitosis, those multipolar, annular, asymmetric and bridge in anaphase (20,21).

Immunohistochemical processing: from the blocks of tissue fixed in formalin and embedded in paraffin, two sections of 4 μm thickness were obtained. To unmask the antigenic epitopes, recovery was performed with sodium citrate solution (pH 6.2) (Borg Decloaker, RTU; Biocare Medical) in a microwave pressure cooker at maximum power for about 5 minutes. Endogenous peroxidases were blocked with 0.9 % hydrogen peroxide for 5 minutes. Tissue sections were incubated with the primary antibodies Ki‑67 (monoclonal; 1:100 dilution, clone MIB-1, Dako, Glostrup, Denmark) and PHH3 (Serine 10) (monoclonal; 1:100 dilution, Novus Biologicals and Bio-Techne brand) for about 45 minutes and then with a biotinylated anti-mouse/anti-rabbit secondary antibody for 30 minutes. To visualize the products of the antigen-antibody reaction, 3,3' diaminobenzidine H2O2 (Dako Corporation, Carpinteria, CA, USA) was used, followed by counterstaining with Harris hematoxylin (Biopack, cod. 0832.08, Argentina). Breast carcinoma samples were used as a positive control for IHC processing. Likewise, as a negative control, IHC processing was performed omitting the incubation step with the primary antibodies.

**Figure 1:**
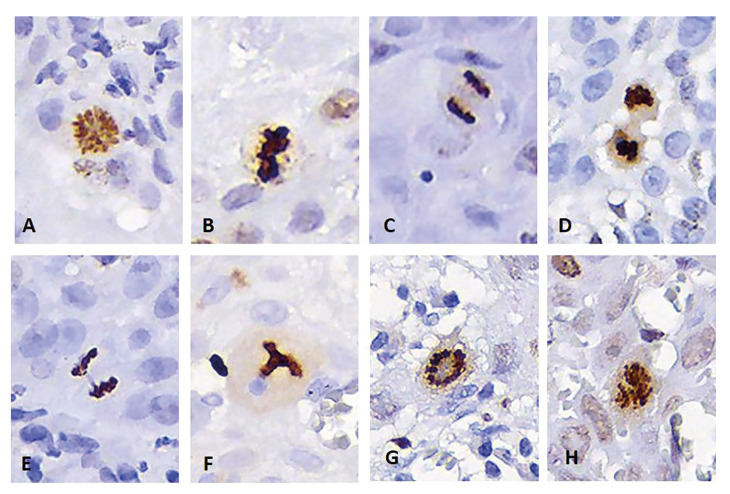
PHH3-positive mitotic figures in OSCC, meeting the criteria for identification: brown nuclear staining, absence of nuclear membrane, and chromatin condensation. A-D Normal mitotic figures: A. Prophase. B. Metaphase. C. Anaphase. D. Telophase. E-H Atypical mitotic figures: E. Anaphasic bridge. F. Tripolar mitosis. G. Ring mitosis. H. Asymmetric mitosis. 40x magnification (A-H).

**Figure 2:**
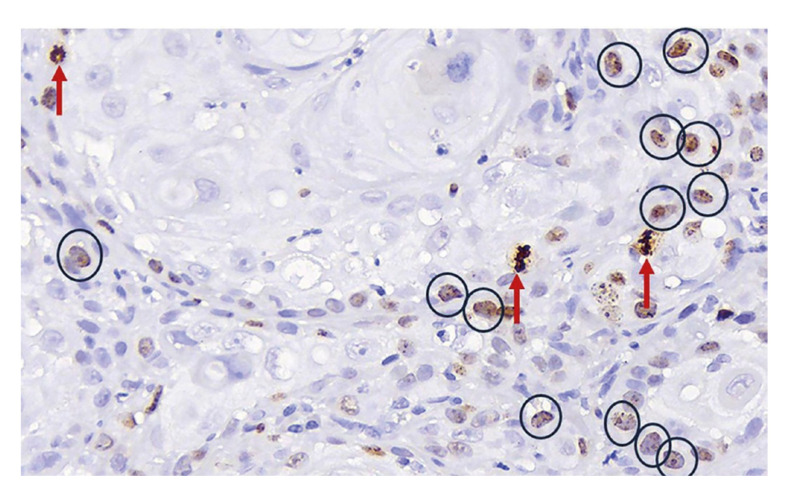
Immunohistochemical microscopic image of PHH3 in OSCC. The mitosis marked with a red arrow correspond to the images considered positive in this study. Nuclei with brown-stained granules and intact nuclear membrane enclosed in circles were not counted, due to not correspond to cells in mitosis. 20x magnification.

IHC evaluation: positive cells for the Ki-67 antibody were considered to be those neoplastic epithelial cells that presented a brown nucleus, regardless of the intensity and pattern of staining (4,9,22). For each of the cases, the degree of positivity for Ki-67 was expressed through the labeling index, expressed as a percentage of positive cells in 1000 tumor cells. PHH3 positive cells were considered those whose nuclei had the following characteristics: a) intense and dense brown staining, b) absence of an intact nuclear membrane, and c) condensed chromatin with normal or atypical MF morphology (9,10,19) (Fig. 1). For the PHH3 antibody, nuclei stained brown, with a smooth, intact nuclear membrane and without conspicuous chromosomal condensation were considered nonspecific and were not included in the immunoquantification (10,19) (Fig. 2). As with Ki-67, the degree of positivity for PHH3 was expressed as a percentage of positive MF in 1000 tumor cells.

Prior to the IHC evaluation, interobserver calibration was performed between two pathologists, who previously agreed on the morphological criteria necessary for the identification of MF and positive cells for biomarkers. The observers carried out their quantifications independently, without knowing the Figures established by each other. The interclass correlation coefficient (ICC) was used to calculate the degree of interobserver agreement (9).

Statistical analysis: in the considered markers comparation, descriptive statistics (mean and standard deviation) were used for categorical variables, and frequency distribution for continuous variables. To compare the expression of the markers according to sex, age, location and histological grade, when it came to two groups, the comparison of means based on the student's t-test for independent samples was used. Likewise, in cases where three or more groups were compared, the analysis of variance (ANOVA) model was used. In cases where a significant association was identified, multiple comparisons were carried out considering the Tukey test. Additionally, scatter diagrams were made to investigate the degree of linear correlation between the three markers, calculating in each case, the Pearson linear correlation coefficient. Survival analysis was carried out using the Kaplan-Meier survival curves. The association of patient survival was analyzed according to the three markers considered, in a multivariate analysis through the Cox proportional hazards model, adjusting the results by sex, age, grouped location and histological grade. While their comparisons were made using the Cox model. All tests were carried out with a significance level of 5%.

## Results

**Figure 3:**
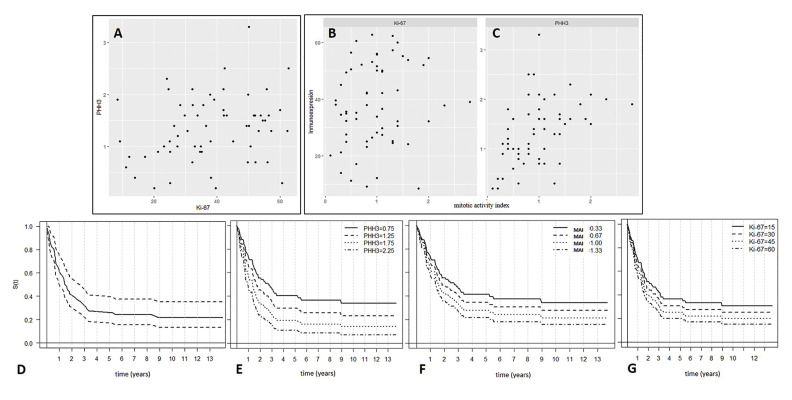
A. Scatter plot showing the existence of a significant but low correlation between PHH3 and Ki-67 (r 0.259 / *p* 0.041). B. Graphic representation showing no significant correlation between Ki-67 and MAI (r 0.167 / *p* 0.194). C. As shown in this diagram, a significant correlation was found between PHH3 and MAI (r 0.450 / *p* < 0.001). D. Kaplan-Meier curve of overall survival. E. Association between survival and PHH3 expression. High expression of PHH3 was significantly associated with a shorter survival period. F. Association between survival and MAI. The increase in MAI was significantly correlated with a shorter survival period. G. Association between survival and Ki-67 expression. High expression of Ki-67 was not significantly associated with a shorter survival period. The Kaplan-Meier E, F, and G curves arise from the Cox proportional hazards model. They are adjusted curves for the different values of the variables.

**Figure 4:**
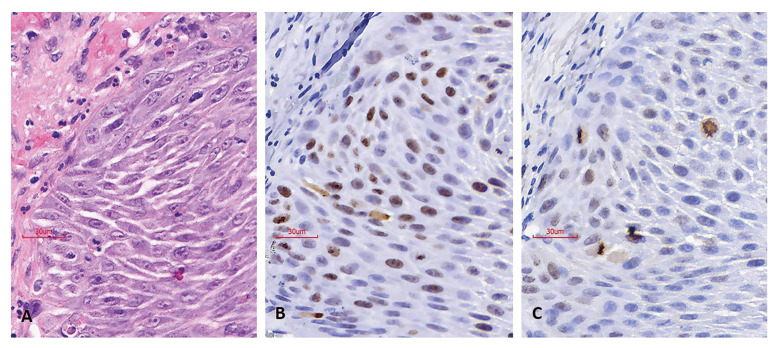
Comparison of the same tumor area in slides stained with H-E and IHC for Ki 67 and PHH3. A. Sheet of H-E where two MF can be identified. B. IHC slide for Ki-67, where several positive cells with nuclear staining of variable intensity are observed. C. IHC slide for PHH3, in which a lower number of positive cells are visualized compared to the IHC slides for Ki-67, identifying four MF that immunoexpress the PHH3 antibody. 20x magnification (A-C).

- Descriptive analysis of the clinical-pathological variables of OSCC: of the 62 cases, the 64.6% corresponded to men and the 59.7% were older than 65 years, the 43.5% were located in others (under this term the located cases were grouped in gingiva, alveolar ridge and extensions to the retromolar trigone and cheek), 25.8 % in tongue, 21.0 % in palate and 9.7 % in the mouth´s floor. Regarding histological grading, 61.3 % corresponded to moderately differentiated OSCC (Grade 2), 27.4 % to well-differentiated OSCC (Grade 1), and only 1.3 % were classified as poorly differentiated OSCC (Grade 3) (Table 1).

- Association between clinical-pathological characteristics and biomarkers Ki-67 and PHH3: a statistically significant relationship was found between the histological grade of OSCC and the immunoexpression of Ki-67 (*p* 0.004) (Table 1). Thus, in the well‑differentiated OSCC, a lower expression of Ki 67 was observed (mean 28.97), rising in the moderately differentiated OSCC (mean 41.27) and reaching the highest expression in the poorly differentiated OSCC (mean 43.37). However, a similar relationship was not found with the PHH3 biomarker (*p* 0.564). Neither could a statistically significant correlation be demonstrated between the biomarkers studied and the independent variables, sex, age and location (Table 1).

- Expression patterns of Ki-67 and PHH3: When correlating the Ki-67 and PHH3 variables, a slight significant relationship was found (*p* 0.041) (Fig. 3). In general, the degree of expression of the PHH3 antibody was markedly lower than that of Ki-67, with a mean of 1.34 (SD 0.62) and 38.14 (SD 14.44), respectively. Additionally, the PHH3 antibody was characterized because in addition to presenting a narrow range of immunostaining (range = 3.1; lower limit = 0.2 and upper limit = 3.3), the intensity of the staining was strong and positively correlated with the morphology of the nuclei according to the different stages of mitosis. In contrast, the Ki-67 antibody demonstrated a wide range of expression (range = 54.2, lower limit = 8.5 and upper limit = 62.7) with variable staining patterns and intensity (Fig. 4).

**Table 1:**
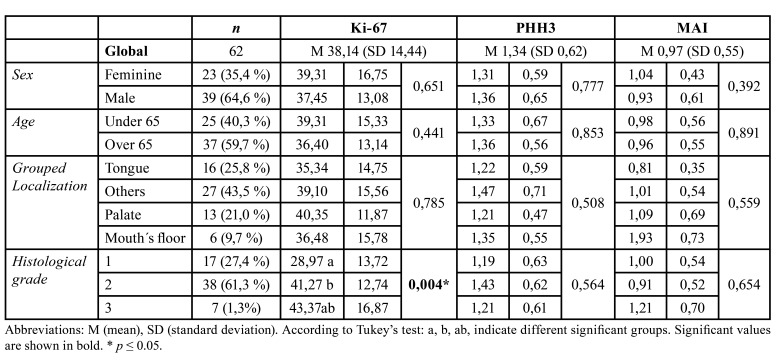
Association between the clinicopathological characteristics of OSCC and the dependent variables: MAI, immunoexpression of Ki-67 and PHH3.

- Survival analysis: the event of death was observed in 48 patients, while 14 patients reached the end of the follow-up period. The survival curve was constructed using the Kaplan-Meier procedure, from which it was estimated that the median overall survival time was 1.51 years (0.92; 2.69) and the survival rate at five years was 27 % (0.18; 0.41) (Fig. 3). Likewise, the study of the possible association between each variable (Ki‑67, PHH3 and MAI) and patient survival was carried out in a bivariate and multivariate manner. From this analysis, in its two forms, it was possible to observe a certain significant association between survival time and PHH3 expression (*p* 0.016) (Fig. 3). A significant and similar relationship is also observed with MAI (*p* 0.031) (Fig. 3), confirming that, at higher values ​​of this, as well as of the immunoexpression of PHH3, the shorter the survival time. In contrast, for KI-67, no statistically significant association was found (*p* 0.295) (Fig. 3). Table 2 shows the results obtained.

**Table 2:**
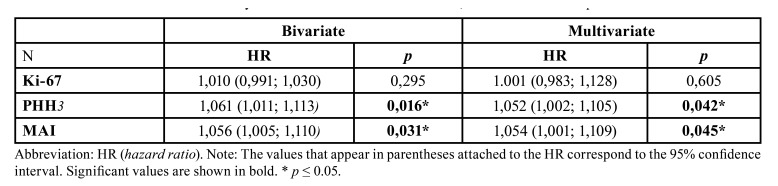
Bivariate and multivariate analysis of the association between KI-67, PHH3 and MAI and patient survival.

When analyzing the relationship between biomarkers and MAI, not enough evidence was found to indicate a significant correlation with Ki-67 (*p* 0.194) (Fig. 3). In contrast, based on the calculated correlation coefficient (r 0.450) with a *p-value* < 0.001, a statistically significant association was observed between MAI and PHH3 (Fig. 3). Likewise, the PHH3 antibody allowed us to more easily identify the positive MF and the fields with the highest mitotic density, thus being able to confirm its role as a specific marker of mitosis. In fact, as shown in Table 3, in 44 cases, the number of mitosis identified by IHC for PHH3 was higher than that obtained based on the recognition of its morphological characteristics in H-E stained slides.

**Table 3:**
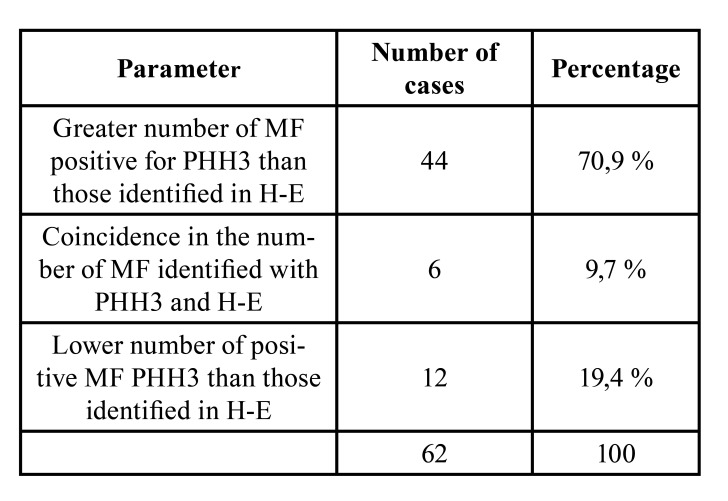
MFs positive for PHH3 in 1000 tumor cells compared to MFs recognized based on their morphological characteristics in H-E stained slides.

## Discussion

The evaluation of cell proliferation in cancer is considered an important histological parameter for defining the biological behavior of the tumor and for determining the individualized prognosis for each patient (7). However, in OSCC, evaluation of tumor proliferation is not part of the current staging system (2). Nevertheless, it constitutes a line of research, like so many others, justified in part by the poor survival rate of diagnosed patients, even in early stages of the disease (4,13). In fact, in this study, the median overall survival time was only 1.51 years (0.92; 2.69) and the five-year survival rate, without considering the stage of the disease, was 27 %. (0.18, 0.41). The low survival rate in our study could be partly because of two reasons. In first place, in Uruguay, the OSCC diagnosis is made in advanced stages, as established in the few publications available (23,24). Second, the sample selection was non-probabilistic for convenience.

The PHH3 antibody, recognized as a biomarker of cell proliferation and specific for cells in mitosis, has been little studied in comparison with other biomarkers of cell proliferation (25). In contrast, in OSCC, unlike the PHH3 antibody, one of the cell proliferation biomarkers that has been widely studied is Ki-67 (4,13,22,26). However, studies on the value of the expression of this biomarker in determining the survival of patients with OSCC have shown contradictory results (3,15). In fact, there was not a statistically significant association between Ki 67 expression and patient survival (*p* 0.295). Along the same lines, the results obtained by Brockton *et al*. and Gonzales Moles *et al*. (27,28). However, it is important to note that recently published research, such as that by Gadbail *et al*. and Jing *et al*. recognize Ki-67 immunoquantification as a reliable prognostic factor in OSCC (4,22). These authors found a significant relationship between the degree of Ki‑67 immunostaining and survival, after studying Ki‑67 expression in large cohorts of 217 and 298 OSCC cases, respectively (4,22). Unlike Ki-67, in this study it was demonstrated a significant relationship between the expression of PHH3 and the survival time of patients with OSCC (*p* 0.016). Although it was previously established in invasive breast and urogenital cancer, it wasn´t found any work in the literature that had previously studied this relationship in OSCC (9,25).

It was also observed a significant relationship between the expression of Ki-67 and PHH3 (*p* 0.041); expected since both are biomarkers of cell proliferation expressed by the fraction of cells that are actively passing through the cell cycle. This association was described in breast cancer by Kim *et al*. and in follicular lymphoma by Bedekovics *et al*.(9,19). In addition, as previously described, it was able to verify a significant relationship between the degree of histological differentiation of OSCC and Ki-67 immunoexpression (*p* 0.004), which supports the potential usefulness of this biomarker in the histopathological classification of OSCC (4,13,22,28). Thus, the least differentiated OSCCs at the histological level are those with the highest number of Ki-67 positive cells (4,13,22,28). However, there was not a similar relationship with the biomarker PHH3 (p 0.564).

When compared the immunostaining patterns of the studied biomarkers, in the Ki-67 slides, nuclear immunostaining was shown with a wide range of intensities, a factor that contributes to low reproducibility (9-11,29). On the other hand, PHH3 showed small variations in the intensity of expression and since only those cells with a strong brown‑stained nucleus and normal or atypical MF morphology should be recognized as positive, in general terms it was easier to identify and quantify (9,10,19). It is important to specify that, as has been observed by other authors, in some cases, the PHH3 biomarker was expressed by cells whose nucleus preserved the nuclear membrane intact and did not present MF morphology, the latter should not be considered in the quantification, for be cells in G2 phase where phosphorylation of histone H3 begins and not cells in mitosis, which do present complete phosphorylation of histone H3 (10,19) (Fig. 2). In addition, as has been previously described in follicular lymphoma and breast cancer, there was a statistically significant association between MAI and PHH3 (*p* < 0.001), expected because the PHH3 antibody is a specific IHC biomarker of MF and because it was used the same quantification criteria, both in the H-E slides for MAI and in those for IHC (9,18,19,21).

The main limitations of the study are the sample size, not including the tumor invasion front as an evaluation parameter because most of the biopsies were incisional, and not having the TNM stage of the cases analyzed.

## Conclusions

The PHH3, is a biomarker of cell proliferation specific to cells in mitosis, with respect to Ki-67, has been underinvestigated in the literature. In this work, a significant relationship was demonstrated between the immunoexpression of the phh3 and the survival of patients with OSCC. A significant association of MAI with survival time was also observed. Regarding the Ki-67, as was previously described in the literature, a positive association was confirmed between the degree of histological differentiation of the OSCC and the marker´s positive immunoexpression. Based on the results obtained, it is important to continue investigating the PHH3 proliferation biomarker in OSCC, with a uniformly standardized IHC protocol and in a large cohort of cases.
